# Mutations in the ENG, ACVRL1, and SMAD4 genes and clinical manifestations of hereditary haemorrhagic telangiectasia: experience from the Center for Osler’s Disease, Uppsala University Hospital

**DOI:** 10.1080/03009734.2018.1483452

**Published:** 2018-09-25

**Authors:** Torbjörn Karlsson, Honar Cherif

**Affiliations:** a Department of Haematology, Uppsala University Hospital, Uppsala, Sweden;; b Center for Osler’s Disease, Uppsala University Hospital, Uppsala, Sweden;; c Department of Medicine, Västmanlands Hospital, Västerås, Sweden

**Keywords:** ACVRL1, ENG, genotype, hereditary haemorrhagic telangiectasia, phenotype, SMAD4

## Abstract

**Aim:** The aim of this retrospective single-centre study was to evaluate whether mutations in the ENG, ACVRL1, and SMAD4 genes were associated with different phenotypes in hereditary haemorrhagic telangiectasia (HHT).

**Methods:** The case records of 21 HHT patients with verified mutations in ENG, ACVRL1, or SMAD4 genes were reviewed. The numbers of HHT diagnostic criteria fulfilled for the three genotypes were compared, as was the prevalence of complications such as iron deficiency anaemia, gastrointestinal haemorrhage, stroke, and cerebral abscess.

**Results:** Our results indicate that mutations in the ENG (HHT1), ACVRL1 (HHT2), and SMAD4 genes result in different HHT phenotypes. Epistaxis debuts earlier and may be more severe in HHT1 than in HHT2. The prevalence of pulmonary arteriovenous malformations (AVM) is higher in HHT type 1, whereas hepatic AVMs are more common in HHT2. One patient with mutations in both ENG and ACVRL1 genes was identified, as were two SMAD4-mutated patients suffering from the overlapping juvenile polyposis-HHT syndrome. Nearly one in five patients in our HHT population has been diagnosed with stroke or cerebral abscess, indicating a high prevalence of cerebral complications.

**Conclusion:** Our results showing that ENG and ACVRL1 gene mutations result in different HHT phenotypes confirm the results from other HHT centres worldwide. Cerebral complications of HHT are common, underscoring the importance of regular screening for pulmonary AVMs and early intervention against such AVMs. We have identified an HHT patient with simultaneous mutations in the ENG and ACVRL1 genes. Surprisingly, this patient has had a mild course of the disease.

## Introduction

Hereditary haemorrhagic telangiectasia (HHT), or Osler–Weber–Rendu syndrome, is a rare autosomal dominant genetic disorder with varying degree of penetrance (1). The overall prevalence of the disease is estimated at 1:10000 (2). The major clinical manifestations of the disorder are the result of development of abnormal vascular structures in different body organs. These include recurrent bleeding from mucosal telangiectasias together with cardiac, cerebral, and hepatic complications caused by arteriovenous fistula formations. The cardinal symptom of HHT is epistaxis, and at least 90% of adult HHT patients suffer from nose bleeds (1). Gastrointestinal (GI) haemorrhage is less common in HHT and usually debuts later in life than epistaxis (3). More than half of HHT patients develop iron deficiency anaemia due to chronic blood loss (4). We have previously shown that anaemic HHT patients who do not respond to iron supplementation can achieve a correction of the anaemia if erythropoietin is added to iron treatment (5). The HHT diagnosis is based on the Curacao criteria which are: recurrent epistaxis, mucocutaneous telangiectasias, evidence of autosomal dominant inheritance, and visceral arteriovenous malformations (AVMs) (6). The HHT diagnosis is definite if three of these criteria are present, suspected if two criteria are present, and unlikely if a patient fulfils fewer than two criteria (7).

Mutations in three different genes, ENG, ACVRL1, and SMAD4, are involved in the pathogenesis of HHT (1). The proteins encoded by these genes belong to the TGF beta superfamily of proteins involved in signal transduction in endothelial cells via the TGF beta cell receptor. All three HHT gene products function in SMAD-dependent signalling pathways in which the activated Smad complex translocates to the cell nucleus and influences transcriptional activation (1). For the HHT genes, most pathogenic variants are functionally null alleles which result in loss of protein expression (2). There are *in vivo* data indicating that loss of function of any of the three HHT genes causes vascular malformation since knockout alleles for ENG, ACVRL1, and SMAD4 all result in HHT phenotypes in mice (1). Molecular testing for ENG, ACVRL1, and SMAD4 mutations is not included in the currently used diagnostic criteria for HHT, but testing is usually performed when there is a strong clinical suspicion of HHT in patients not fulfilling the diagnostic criteria, and for testing family members of HHT patients. Mutations in the ENG and ACVRL1 genes result in different HHT phenotypes, commonly referred to as HHT1 and HHT2, respectively (8–12). For example, it is more common to find pulmonary AVMs (PAVMs) in HHT1, but hepatic AVMs (HAVMs) are more common in HHT2. There are conflicting data published regarding age at debut and severity of epistaxis comparing HHT type 1 and type 2 (9,10,12). Approximately 80% of all HHT patients have ENG- or ACVRL1-mutated genes, but only a few per cent carry SMAD4 mutations (1). Mutations in the SMAD4 gene can give rise to a combined syndrome of HHT and juvenile polyposis (JP), the JP-HHT syndrome (13). JP-HHT patients exhibit symptoms of HHT and JP in addition to an increased risk for GI cancer (13–15). Several hundred different mutations, including deletions, insertions, missense, and nonsense mutations, have been identified in the HHT-causing genes, and most of them have been reported only once (1). For an overview of all currently reported ENG and ACVRL1 mutations in HHT, see https://www.hhtmutation.org.

PAVM can cause bypass of emboli and septic material with risk of stroke or brain abscess. Due to these risks, HHT guidelines commonly recommend a chest computed tomography (CT) at diagnosis (or when the patient is 18 years old) and every fifth year thereafter (4,16). Therapeutic embolization is the treatment of choice for significant PAVM. Screening for HAVM is recommended only if a HHT patient develops liver disease (4,16) or high-output cardiac failure (17,18). Approximately 10% of all HHT patients develop cerebral AVM (CAVM), but we do not screen for these in asymptomatic patients, since the therapeutic alternatives for this kind of AVM are limited and intervention is not without risk (4,16).

The aim of this retrospective study was to evaluate whether mutations of the ENG, ACVRL1, and SMAD4 genes were associated with different HHT phenotypes in our patient population. The numbers of diagnostic criteria fulfilled for the different genotypes were compared, as was the prevalence of complications such as iron deficiency anaemia, gastrointestinal haemorrhage, stroke, and cerebral abscess.

## Material and methods

In this study we identified adult (≥18 years of age) HHT patients and patients with suspected HHT who were referred to the Center for Osler’s Disease, Uppsala University Hospital, for ENG, ACVRL1, and SMAD4 gene mutation testing during 2010–2017. The case records of the patients were reviewed, and relevant data were collected including results from the mutation analyses. The genetic analyses were performed at Invitae, San Francisco, CA, USA; Institut fur Humangenetik, UKB, Bonn, Germany; Labor Lademannbogen MVZ, Hamburg, Germany; UMC Utrecht, Utrecht, The Netherlands; and PreventionGenetics, Marschfield, WI, USA. Iron deficiency anaemia was defined as a haemoglobin value less than 130 g/L and 120 g/L in men and women, respectively, in combination with ferritin less than 25 µg/L in men and 10 µg/L in women. Patients were diagnosed with iron deficiency anaemia if they at least once had met these criteria for anaemia. An endoscopically verified bleeding lesion was required for a patient to be diagnosed with GI haemorrhage. Due to the small number of patients the results are expressed as means (range). This study was approved by the Ethics Committee of Uppsala (# 2017/242) and performed in accordance with the Declaration of Helsinki. Written informed consent was obtained from all patients.

## Results

In our patient population of 21 patients with genetically verified HHT, 5 (24%) and 13 (62%) were diagnosed with HHT1 and HHT2, respectively. One patient (5%) had mutations in both ENG and ACVRL1 genes (HHT1 + 2), and we identified two (9%) patients with SMAD4 mutations. In the HHT1 group two patients were first-degree relatives, whereas the other three were not related to each other. In the HHT2 group seven patients were not relatives, and there were three pairs of first-degree relatives. The ENG mutations identified were c.68-1G>C (two cases), c.392delC, and c.219 + 5G. For one of the ENG-mutated patients the type of mutation was not reported. The ACVRL1 mutations were c.41dupT (two cases), large deletion in exon 9 (two cases), c.1378-1G>A, c.1120C>T (two cases), c.129delG (two cases), c.889T>C, c.430C>T, and c.1246 + 2T>C. One patient was diagnosed with a mutation in the ACVRL1 gene, but the type of mutation was not reported. One of the patients in the SMAD4 group had a c.1148T>G mutation, whereas the type of mutation in the other SMAD4 patient was not reported.

The median (range) number of diagnostic criteria fulfilled for the entire HHT population was 3 (range 2–4), whereas it was 4 (range 2–4) and 3 (range 2–4) for the HHT1 and HHT2 populations, respectively. Four (19%) of the 21 patients did not have any first-degree relative diagnosed with HHT, and none of them belonged to the HHT1 group (Table 1).

**Table 1.** Percentage of the total HHT population and the subpopulations fulfilling the four different Curacao criteria for HHT.

Variable (%)	Total HHT (*n* = 21)	HHT1 (*n* = 5)	HHT2 (*n* = 13)	HHT1 + 2 (*n* = 1)	SMAD4 (*n* = 2)
Heredity	81	100	85	0	50
Epistaxis	95	100	92	100	100
Telangiectasia	90	80	100	100	50
AVM	43	60	31	0	100

AVM: arteriovenous malformation; HHT: hereditary haemorrhagic telangiectasia.

**Figure 1.**
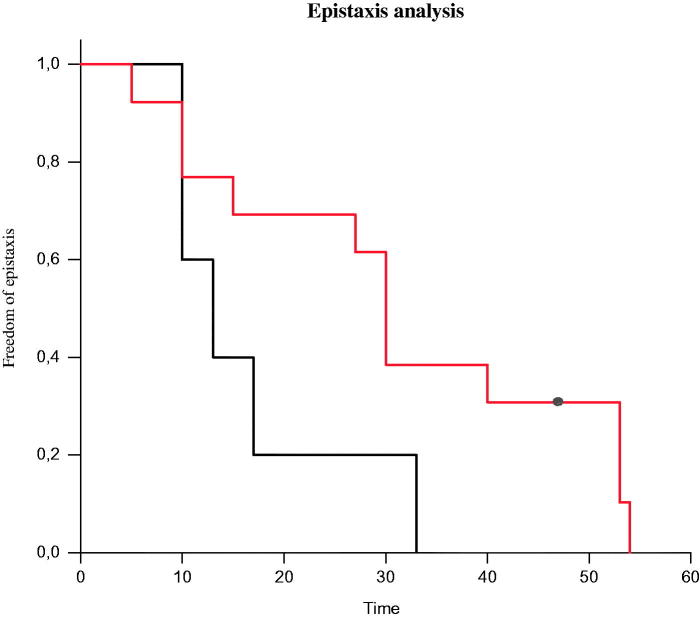
Kaplan–Meier survival curve for the HHT1 and HHT2 groups, respectively, showing the probability of remaining free of epistaxis. HHT1, black line; HHT2, red line. HHT: hereditary haemorrhagic telangiectasia.

The median (range) age of debut of epistaxis was 13 (range 10–33) years for the HHT1 group, whereas it was 30 (range 5–54) years for the HHT2 group. At the age of 33 years the penetrance for epistaxis was 100% in the HHT1 group (Table 1), and at the same age it was 62% in the HHT2 group. At the age of 50 years the penetrance for epistaxis was still incomplete in the latter group. For the three patients who did not belong to the HHT1 or HHT2 group the penetrance for epistaxis was 100% at the age of 40 years. Figure 1 shows a Kaplan–Meyer survival plot of the chance of being free from epistaxis for the HHT1 and HHT2 groups, respectively. Over a two-year period (January 2016 to December 2017) the mean number of endonasal laser coagulation procedures for epistaxis was 0.7 and 0.35 per patient and year for the HHT1 and HHT2 groups, respectively. Ninety per cent (19/21) of all HHT patients in this study suffered from mucocutaneous telangiectasias. Table 1 shows the percentage of patients fulfilling the four different Curacao criteria for the entire HHT population and the HHT1, HHT2, HHT1 + 2, and SMAD4 subpopulations, respectively.

Ninety-five per cent (20/21) of the HHT patients had been screened radiologically at least once for PAVM. In the HHT1 group PAVM was present in 60% (3/5) of the patients, whereas only one patient (8%) in the HHT2 group was diagnosed with PAVM. HAVM was detected in 5/13 (38%) of the patients in the HHT2 group, whereas no such AVM was detected in the HHT1 group (Table 2). One of the HHT2 patients developed HAVM-associated terminal liver failure and was subjected to a liver transplantation at the age of 46 years. He was alive 14 months after the transplantation. None of the patients in the HHT1 or HHT2 groups was diagnosed with CAVM. Both patients carrying SMAD4 mutations suffered from PAVM and HAVM, and one of them also from CAVM (Table 2). One patient in our HHT cohort exhibited mutations in both the ENG (c.572G>A) and ACVRL1 (c.673_674delAG) genes. Since this patient did not have any first-degree relative with HHT symptoms, these genetic aberrations are probably *de novo* mutations. This individual, who fulfilled two HHT diagnostic criteria, had started to suffer from nose bleeds at the age of 40 years. He had not developed any AVM, but he had mucocutaneous telangiectasias. Both patients with SMAD4 mutations suffered from nose bleeds and had multiple AVMs, but only one of them had a first-degree relative with HHT. These two patients both suffered from the overlapping JP-HHT syndrome, and they had been diagnosed with colorectal and thyroid cancer, respectively.

**Table 2.** Percentage of the total HHT population and the subpopulations diagnosed with PAVM, HAVM, CAVM, IDA, and GI haemorrhage.

Variable (%)	Total HHT (*n* = 21)	HHT1 (*n* = 5)	HHT2 (*n* = 13)	HHT1 + 2 (*n* = 1)	SMAD4 (*n* = 2)
PAVM	29	60	8	0	100
HAVM	33	0	38	0	100
CAVM	5	0	0	0	50
IDA	62	60	62	100	50
GI haemorrhage	10	0	15	0	0
Stroke/brain abscess	19	40	8	0	50

CAVM: cerebral arteriovenous malformation; GI: gastrointestinal; HAVM: hepatic arteriovenous malformation; HHT: hereditary haemorrhagic telangiectasia; IDA: iron deficiency anaemia; PAVM: pulmonary arteriovenous malformation.

In total 62% of the HHT patients were diagnosed with iron deficiency anaemia, and there appears to be no difference in anaemia prevalence comparing the HHT1 and HHT2 groups (Table 2). Two cases of endoscopically verified bleeding telangiectasias of the GI tract were recorded in our HHT material. Both of these were HHT2 patients. Out of the 21 HHT patients in our material, four (19%) were diagnosed with an HHT-related central nervous system lesion (Table 2). Two of the HHT1 patients were diagnosed with stroke, and two other patients had been treated for brain abscess.

## Discussion

In this retrospective single-centre study, performed at the Center for Osler’s Disease at Uppsala University Hospital, the aim was to evaluate whether mutations in the ENG, ACVRL1, and SMAD4 genes were associated with different HHT phenotypes in our patient cohort. The prevalence of HHT-related complications such as iron deficiency anaemia, GI haemorrhage, stroke, and brain abscess was also evaluated. Such evaluations have previously been reported from other HHT centres (8–12), but this is the first report on genotype–phenotype association in a Swedish HHT patient population.

Five of the 21 (24%) patients in our HHT population were diagnosed with HHT1, whereas 13 (62%) had HHT2. In the study by Lesca and co-workers the distribution between HHT1 and HHT2 was similar to what we report here (12). This contrasts with what has been reported from two other HHT centres (9,10). For the HHT population in total, 81% of the patients have reported a family history of the disease. Since this retrospective study was restricted to patients who had been visiting our clinic, their families were not systematically tested for gene mutations or evaluated for HHT symptoms; it is therefore possible that less than 19% of the cases were caused by *de novo* mutations. The clinical symptoms of HHT can be extremely varied even in family members sharing the same gene mutation (1), so we cannot exclude that the four patients without any obvious heredity belonged to families with undiagnosed HHT with mild symptoms of the disease. Sporadic cases are considered rare in HHT, but a high rate of SMAD4 *de novo* mutations has previously been reported in the JP-HHT syndrome (13,14). We found that the median age of debut of epistaxis was more than twice as high (33 versus 13 years) and the penetrance of epistaxis incomplete in the HHT2 group compared with the HHT1 group. This difference in the age of debut of epistaxis has been reported previously, as has the incomplete penetrance of epistaxis in HHT2 (12). Since we could not properly assess the severity of epistaxis in individual patients from the case records, we have tried to assess this indirectly by comparing the number of endonasal laser coagulation interventions for epistaxis. Endonasal laser coagulation is the commonly used therapy for chronic and severe epistaxis in HHT (4). The number of this kind of intervention was obtained from the patient case records. The mean number of endonasal laser coagulation procedures for epistaxis per patient and year was twice as high in the HHT1 group as in the HHT2 group, possibly indicating that epistaxis is more severe in HHT1.

The prevalence of any AVM was 60% and 31% in the HHT1 and HHT2 groups, respectively. This difference in AVM prevalence is primarily explained by the higher prevalence of PAVM in the HHT1 group (60% versus 8%). HAVM was found in 38% of the HHT2 patients in our patient cohort. This prevalence is in line with what others have reported (10–12). Table 3 summarizes the prevalences of PAVM, HAVM, and CAVM in HHT1 and HHT2, respectively, in six studies published. The prevalence of symptomatic liver disease is less than 10% in HHT (19). HHT patients with HAVM-associated liver disease or high-output cardiac failure who do not respond to maximal medical therapy are recommended liver transplantation, which has a curative potential for HAVM-associated liver disease (19,20). Indeed, one HHT2 patient in our patient population has successfully undergone a liver transplantation.

**Table 3.** Percentage of vascular malformations (PAVM, HAVM, and CAVM) in HHT1 (A) and HHT2 (B) in the six different studies published on genotype–phenotype correlation in HHT.

Type of AVM (%)	Present study	Berg et al. (8)	Kjeldsen et al. (9)	Bayrak-Toydemirv et al. (10)	Letteboer et al. (11)	Lesca et al. (12)
A: HHT1						
Pulmonary	60	35	46	59	49	59
Hepatic	0	5	NR	2	8	2
Cerebral	0	8	2	16	NR	16
B: HHT2						
Pulmonary	8	0	13	39	5	29
Hepatic	38	6	NR	28	41	28
Cerebral	0	3	4	2	NR	2

AVM: arterio-venous malformation; CAVM: cerebral AVM; HAVM: hepatic AVM; HHT: hereditary haemorrhagic telangiectasia; NR: not reported; PAVM: pulmonary AVM.

The patient with mutations in both ENG and ACVRL1 genes reported here had not developed any AVM. He had a benign course of the disease with no need for endonasal laser coagulation intervention for epistaxis and no history of GI haemorrhage. To our knowledge this is the first report describing a patient with mutations in both ENG and ACVRL1 genes.

Both SMAD4-mutated patients had been diagnosed with PAVM and HAVM. One of the SMAD4 patients had also been diagnosed with a CAVM. This was the only patient in our patient population with such an AVM.

In a report by Bayrak-Toydemir and co-workers (10) the prevalence of iron-deficiency anaemia in HHT was over 50%, consistent with our finding of an anaemia prevalence of approximately 60%. Although the prevalence of migraine (8) and cerebral abscess (12) is higher in HHT than in the general population, there are limited data published on the prevalence of PAVM-related cerebral complications in general in HHT. Here we report a high prevalence of cerebral complications in our HHT population with nearly one in five patients diagnosed with such complications in the form of stroke or cerebral abscess.

Two of the patients reported here were diagnosed with the JP-HHT syndrome. In general, patients with SMAD4 mutations display a full range of HHT clinical features. For example, in one study approximately 70% of the patients with SMAD4 mutations were diagnosed with PAVM, and at least as many suffered from nose bleeds (14). Interestingly, identical mutations in the SMAD4 gene can cause JP in some patients and JP-HHT in others (14). Both of our JP-HHT patients were diagnosed with GI polyps, one of them with colorectal cancer, whereas the other one had thyroid cancer. SMAD4 mutations are involved in the pathogenesis of JP (21), and it has been shown that mutations in this gene also contribute to tumour progression in thyroid cancer (22,23).

In summary, in the first study on the genotype–phenotype correlation in HHT from a Swedish HHT centre we report results indicating that epistaxis debuts earlier and may be more severe in HHT1 than in HHT2. The prevalence of PAVM is higher in HHT1 than in HHT2, whereas HAVM prevalence is higher in HHT2. An HHT patient with mutations in both ENG and ACVRL1 is also described. In addition, we report a high prevalence of cerebral complications in our HHT patient cohort. Nearly one in five of the patients has been diagnosed with ischaemic stroke or cerebral abscess.
